# Maternal interpersonal problems and attachment security in adolescent offspring

**DOI:** 10.1186/s40479-022-00188-8

**Published:** 2022-07-01

**Authors:** Sophie Kerr, Francesca Penner, Gabrielle Ilagan, Lois Choi-Kain, Carla Sharp

**Affiliations:** 1 Department of Psychology, University of Houston, 3695 Cullen Blvd, Room, Houston, TX 126 USA; 2 Child Study Center, Yale University, New Haven, CT USA; 3 Department of Psychology, Fordham University, Bronx, NY USA; 4 Gunderson Personality Disorders Institute, McLean Hospital, Belmont, MA USA; 5 Harvard Medical School, Department of Psychiatry, Cambridge, MA USA

**Keywords:** Interpersonal problems, Attachment, Intergenerational transmission, Adolescent attachment

## Abstract

**Background:**

Research on parent-level factors linked to adolescent attachment security would inform interventions to prevent or reduce youth psychopathology and other negative outcomes. The current study examined one relevant parent-level variable: maternal interpersonal problems. Interpersonal problems, a key characteristic of personality pathology, are well described by the interpersonal circumplex (IPC) and have been shown to be associated with maladaptive adult attachment in close/romantic relationships; however, studies have not examined relationships with offspring attachment. Therefore, the first aim of the current study was to examine the relationship between maternal interpersonal problems and adolescent attachment insecurity. Based on previous evidence that parents’ recalled bonding with caregivers is associated with the quality of bonding and attachment with offspring, the second aim was to examine whether mothers’ recalled bonding with their own mothers partially explained this relationship.

**Methods:**

Participants included 351 psychiatric inpatient adolescents (M_age_ = 15.26, 64.1% female) and their biological mothers. Logistic regressions tested whether maternal interpersonal problems were associated with Child Attachment Interview classifications (secure vs. insecure; secure vs. preoccupied vs. dismissing; not disorganized vs. disorganized). A mediation model (*N* = 210) tested whether the relationship between maternal interpersonal problems and adolescent attachment was mediated by the mother’s recalled maternal bonding.

**Results:**

Maternal interpersonal problems were associated with insecure (vs. secure), dismissing (vs. secure), and preoccupied (vs. secure) attachment. There was no significant relationship between maternal interpersonal problems and disorganized attachment. Mediation analyses showed that maternal interpersonal problems were indirectly related to adolescent attachment security via the mother’s recalled maternal care, though only a small amount of variance (7%) in adolescent offspring attachment was accounted for by the model.

**Conclusions:**

Results provide the first evidence that maternal interpersonal problems are associated with higher likelihood of insecure attachment in adolescents. Therefore, researchers could consider drawing upon the IPC literature to further examine mechanisms of intergenerational risk and to tailor interventions aimed to improve parent-child relations and attachment. Additionally, findings highlight the mediating role of the mothers’ recalled experiences with caregivers in the transmission of risk, suggesting attachment-based or mentalization-based interventions may be helpful for mothers with interpersonal problems and personality pathology.

**Supplementary Information:**

The online version contains supplementary material available at 10.1186/s40479-022-00188-8.

## Background

Attachment security is a significant factor for socio-emotional development and mental health in youth, with meta-analytic studies indicating that insecure attachment is associated with internalizing psychopathology [18], externalizing psychopathology [14], and poorer social competence [17] in children. Parent-child attachment security continues to play an important role through adolescence, which is thought to be the second most critical developmental window after infancy and early childhood [41]. Social, emotional, and neurobiological changes during adolescence present different challenges for youth and parents as well as new threats to secure attachment [32, 33, 66], highlighting the importance of studying attachment during adolescence specifically. Additionally, there is increased risk for psychopathology during adolescence [49] and research shows associations between attachment and adolescent mental health [39]. For example, one study found that secure attachment acts as a protective factor against adolescent borderline personality disorder (BPD) through enhanced positive emotion regulation [30]. Therefore, identifying risk factors for insecure attachment during adolescence may add to knowledge on etiology of adolescent mental disorders. Moreover, elucidating parent-level factors linked to attachment during adolescence can inform interventions with parents to prevent and reduce youth psychopathology. For example, studies have shown that maternal anxiety, depression, and borderline personality disorder are linked with child or adolescent attachment insecurity [10, 12, 23]. The current study focuses on the relationship between adolescent attachment and one parent-level variable: maternal interpersonal problems.

**Fig. 1:**
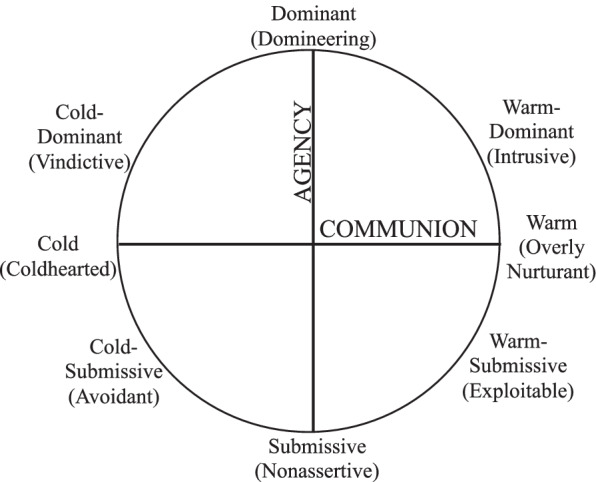
The Interpersonal Circumplex

Maternal interpersonal problems, a construct highly related to personality dysfunction and personality pathology, may be a particularly relevant parental variable related to offspring attachment, yet this has not been studied. Contemporary interpersonal theory [46] defines and measures interpersonal functioning using the interpersonal circumplex (IPC) model [70]. As shown in Fig. 1, the IPC organizes interpersonal behavior on two axes: agency and communion. Measures of the IPC, including the widely used self-report Inventory of Interpersonal Problems (IIP; [2, 27]), operationalize severity as greater interpersonal problems on these two dimensions. Visually, severity is represented as distance from the center of the circle (see Fig. 1). Decades of IPC research has focused on the conceptualization and treatment of personality pathology [29, 73] and recent evidence supports that the IPC captures personality pathology as defined by the DSM-5 Section III Alternative Model of Personality Disorders (AMPD; [45, 75]). In contrast to categorical models of personality pathology, the interpersonal perspective has the advantage of being a process-oriented clinical framework [26]. Additionally, there is an extensive and methodologically rigorous literature on the IPC and psychotherapy processes (for a review, see [47]) as well as empirically supported methods for applying the IPC and interpersonal theory to psychotherapy [9, 74]. Demonstrating a link between parental interpersonal problems and attachment in offspring would support drawing upon the IPC literature to adapt and refine interventions to prevent the intergenerational transmission of personality problems and psychopathology.

Researchers have examined how interpersonal problems impact attachment relationships and behaviors, but all studies have been limited to adult attachment in close or romantic relationships. Evidence suggests that greater interpersonal problems on measures of the IPC are associated with greater attachment avoidance, anxiety, and insecurity in close relationships in clinical [19, 31, 37] and nonclinical samples [5, 69]. A recent systematic review of studies reporting correlations between interpersonal problems and attachment anxiety/avoidance in close adult relationships over the past 15 years concluded that adult interpersonal problems are strongly associated with both attachment domains, with correlations ranging from .25–.60 for attachment anxiety and .24–.63 for attachment avoidance [22]. Based on findings that adults with interpersonal problems struggle to form adaptive attachments in close and romantic relationships, we hypothesized that mothers with high levels of interpersonal problems would also struggle in attachment relationships with their children, resulting in insecure offspring attachment; however, this has not yet been examined.

In considering potential pathways explaining the relationship between parental interpersonal functioning and offspring attachment, the way that parents recall their experiences with their caregivers is likely impacted by their own interpersonal functioning and may impact the relationship that they build with their children. This idea is supported by substantial evidence that parents’ recollections of bonding with their own caregivers is associated with the quality of bonding and attachment they have with their offspring. For example, an association between parent and offspring reports of bonding (i.e., experiences of affection and nurturing or fostering of autonomy) with caregivers as measured by the Parental Bonding Inventory (PBI; [43]) was demonstrated in mothers and daughters over 10-year follow-up while controlling for depression, temperament, and socioeconomic status [40], as well as in adults with drug use problems and their parents [64]. Studies have also shown that recalled bonding with parents predicts attachment-related feelings and behaviors with fetal or infant offspring. For example, mothers’ recalled bonding with their own mothers predicted reported attachment to their baby during pregnancy and observed attachment behaviors in the 2 days following childbirth [62]. Similarly, Handelzalts et al. [21] demonstrated associations between recalled maternal bonding and maternal-fetal attachment during pregnancy and Hall et al. [20] found that mothers’ recalled parental bonding was associated with reported attachment and bonding to their infant at 1 month and 6 months postpartum.

Given evidence that their recollections of bonding with caregivers is associated with how parents build relationships with their own children, we hypothesized that recalled experiences with caregivers would partially explain (i.e., mediate) the relationship between parental interpersonal problems and insecure attachment with offspring. In explaining the rationale for this model, we emphasize that we measured retrospective reporting of maternal bonding, not actual experiences of bonding. Recalling caregiving experiences requires the individual to draw upon their current representation of their caregiver, meaning that recalled maternal bonding is conceptualized as a proximal factor. This idea is supported by findings that recollections of adverse childhood experiences are more closely related to current psychopathology than actual experiences of adverse childhood experiences, measured prospectively, in the absence of recollections of the adversity [4, 11].

Based on this background, the first aim of the current study was to examine associations between maternal interpersonal problems and attachment security reported by adolescent offspring. Given evidence that interpersonal problems are associated with maladaptive attachment in close/romantic relationships, we hypothesized a priori that this would extend to the parenting context and that maternal interpersonal problems would be associated with insecure attachment reported by offspring. Based on evidence that recollections of bonding with caregivers is associated with how parents interact with and relate to their own children, the second aim was to test whether parents’ recalled bonding with caregivers mediates this relationship. We hypothesized a priori that the relationship between maternal interpersonal problems and adolescent attachment would be mediated by the mother’s own recalled maternal bonding.

## Methods

### Participants and procedures

The sample consisted of adolescents recruited from an inpatient unit at a private psychiatric hospital in a large metropolitan city in the Southwestern United States and their mothers. The current study is a secondary analysis of data drawn from a larger study. The methods and protocol of the larger study are described in detail by Sharp et al. [57]. For the larger study, inclusion criteria were proficiency in English and admission to the adolescent inpatient unit and exclusion criteria were a diagnosis of an autism spectrum disorder, schizophrenia or other psychotic disorder, active mania, or IQ below 70. Given the aims of the current study, we only included mother-child dyads with mothers who completed the measure of interpersonal problems, the Inventory of Interpersonal Problems-Short Circumplex Form (IIP-SC), and offspring who completed the Child Attachment Interview. Additionally, we excluded adopted adolescents from current analyses because we lacked sufficient information regarding the timing and nature of the adoption to account for possible differences in the caregiving context. Of 653 consecutive admissions of adolescents who met eligibility requirements and consented to participation, we excluded 131 adolescents who had parent reports completed by their fathers and 70 who had parent reports completed by an adoptive mother. Of the remaining 461 dyads, 351 dyads completed the IIP-SC and CAI. Therefore, our final sample consisted of 351 biological mother-adolescent dyads who completed these measures.

Adolescents included 225 females (64.1%) and ranged in age from 12 to 17 (*M* = 15.26, *SD* = 1.45). The majority of adolescents were white and not Hispanic (85.5%, *n* = 294), 6.6% were Hispanic/Latinx (*n* = 23), 1.1% (*n* = 4) were Black/African American, 1.1% were Asian (*n* = 4), and 5.4% (*n* = 19) were multiracial or another race or ethnicity. Of the 305 (86.9%) parents who reported their marital status, most (73.1%) parents were married, 20.4% were separated or divorced, 3.3% were widowed, 2.3% were living with someone as if married, and 1.0% were never married. Of these 305 mothers, 32.8% reported holding a graduate degree, 48.5% reported a bachelor’s degree, 16.8% reported completing some college or an associate degree, and 1.9% reported some high school or a high school diploma. Of the 268 (76.4%) parents who reported their annual household income, 53.4% reported earning $200,000 or more, 24.6% reported earning between $100,000–$200,000, 11.57% reported earning between $50,000–$100,000, and 7.61% reported earning less than $50,000. Adolescents had the following break-down of DSM-IV diagnoses: 58.4% met criteria for a depressive disorder, 63.7% for an anxiety disorder, 42.5% for an externalizing disorder, 33.6% for borderline personality disorder, 8.7% for an eating disorder, and 7.8% for a bipolar disorder. The average length of stay in the inpatient unit was 35.31 days (SD = 12.53).

For the second aim of this study, examining whether mothers’ own recalled maternal bonding mediated the relationship between maternal interpersonal problems and offspring attachment, we used a subsample of 210 of the 351 participants whose mothers had participated in the study while the Parental Bonding Inventory (PBI) was included in the battery. This subsample of participants did not differ from the remainder of the sample in gender (*p* = .53), age (*p* = .39), race or ethnicity (*p*s > .16), or in diagnostic characteristics (*p*s > .26), except that the subsample had lower rates of DSM-IV depressive disorders (47.8%, *p* < .001) and anxiety-related disorders (58.6%, *p* < .05).

To participate in the study, parents first provided consent and adolescents provided assent. All consecutive admissions to the adolescent inpatient unit were approached for participation. Assessments were conducted by clinical psychology doctoral students or trained research coordinators during the first 2 weeks of the adolescent’s admission. The study protocol was approved by the appropriate institutions’ human subjects review committees.

### Measures

#### Interpersonal problems in mothers

The Inventory of Interpersonal Problems-Short Circumplex Form (IIP-SC; [59]) is a 32-item measure corresponding to the IPC. It is a short form of the IIP-Circumplex Form [2], which was based on the original IIP [27]. Participants are asked about distressing interpersonal behaviors that they find “hard to do” (e.g., “It is hard for me to feel close to other people”) or “do too much” (e.g., “I try to please other people too much”). Items are rated are on a five-point Likert scale ranging from 0 (not at all) to 4 (extremely). Scale scores can be derived for each of the eight octants of the IPC (Domineering, Intrusive, Overly-Nurturant, Exploitable, Non-Assertive, Socially Avoidant, Coldhearted and Vindictive) by summing their 4 corresponding items, and a total score is calculated by summing all items. In the present study, we used the *T*-score for total scale, with higher scores reflecting greater overall interpersonal problems. Internal consistency reliability, test-retest reliability, and validity of the IIP-SC have been demonstrated [25, 59]. In the present study, the Cronbach’s alpha for the IIP-SC was α = .90.

#### Adolescent attachment security

The Child Attachment Interview (CAI; [63]) is a semi-structured interview that assesses mental representations of attachment relationships consistent with clinical theories of attachment originally proposed by Bowlby [7] and Ainsworth [1]. The interview was originally adapted from the Adult Attachment Interview and developed for use with 8- to 12-year-old children but has been used and evaluated in adolescents (see Privizzini, [48] for a review). The interview consists of 17 questions designed to elicit self-representations and representations of primary attachment relationships. Children are assessed on their ability to describe their attachment experiences coherently and collaboratively and to reflect on these experiences and their impact on them. For the current study, interviews were conducted in private and video recorded, then transcribed and coded by an independent coder. To become certified coders, doctoral-level clinical psychology students and trained clinical research assistants were first required to undergo training and show 80% reliability on a set of videos with the CAI authors. Based on the CAI narrative, raters assign a value from 1 to 9 (1 = absence of the construct, 9 = high level on the construct) on each of the 9 subscales: emotional openness, balance of positive and negative reference to attachment figures, use of examples, preoccupied anger, idealization, dismissal, resolution of conflicts, and overall coherence. Using the distributions of these subscale scores and behavioral analysis of the interview, the rater then determines the child’s attachment classification (secure, insecure-dismissing, insecure-preoccupied) with each caregiver. Children are also classified on attachment disorganization (present or absent), which is independent of the secure/dismissing/preoccupied classification and is assigned when there is strange, dysregulated, dissociated, or controlling behavior. Attachment security classifications can be analyzed as two-way secure vs. insecure (encompassing preoccupied and dismissing), three-way secure vs. dismissing vs. preoccupied, and not disorganized vs. disorganized. For the current study, we analyzed all three of the attachment classifications.

Across clinical and community samples of children and adolescents, the CAI has shown adequate interrater reliability, test-retest reliability, concurrent validity with the Separation Anxiety Test, self-report measures of attachment, parental care, and parental warmth and anger, and discriminant validity from demographic (age, SES, ethnicity) and cognitive variables (IQ, expressive language), and temperamental characteristics [6, 55, 58, 66]. In the current sample, interrater reliability was assessed in a randomly selected 62 (17.7%) of the interviews that were coded by additional raters. Interrater reliability was found to be moderate for the two-way secure vs. insecure classification (*κ* = .46, *p* < .001, 77.4% agreement), the secure vs. dismissing vs. preoccupied classification (*κ* = .41, *p* < .001, 63.1% agreement), and the not disorganized vs. disorganized classification (*κ* = .47, *p* < .001, 88.7% agreement).

#### Mother’s recalled parental bonding

The Parental Bonding Instrument (PBI; Parker et al., 1979) is a 25-item retrospective self-report of recalled parental attitudes and behaviors. Items are rated on a four-point Likert scale ranging from “very like” to “very unlike.” The 12-item Care subscale assesses recalled affection and nurturing from a caregiver, e.g., “Spoke to me in a warm and friendly voice.” The 13-item Overprotection subscale assesses recalled control and the fostering of autonomy by a caregiver, e.g., “Tried to control everything I did.” Higher scores indicate higher levels of recalled caring and overprotective behaviors, respectively. The PBI can be used to assess recalled maternal or paternal care. For the current study, we only analyzed recalled maternal care. The PBI has shown satisfactory construct and convergent validity [44], and acceptable reliability [71]. In the current study, the Cronbach’s alpha was α = .94 for the care subscale and α = .86 for the overprotection subscale.

### Data analytic approach

Analyses were conducted using SPSS Version 26 and MPlus Version 8. Preliminary analysis, including descriptive statistics and Pearson correlations, were first calculated. To address Aim 1, binary logistic regressions were used to evaluate relationships between maternal interpersonal problems and adolescent attachment on the CAI (two-way secure vs. insecure rating, and two-way not disorganized vs. disorganized). A multinomial logistic regression evaluating relationships with the three-way secure vs. dismissing vs. preoccupied classification is in the supplementary materials. T-tests and one-way analysis of variance (ANOVA) were used to examine bivariate relationships with possible covariates of age and gender and between other main study variables.

**Fig. 2:**
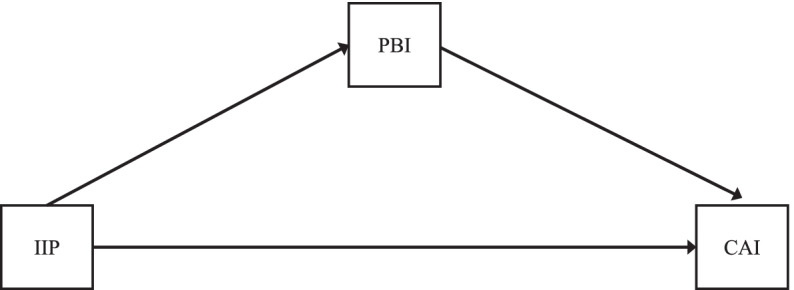
Hypothesized mediation models with maternal interpersonal problems (IIP-SC Total), mothers’ recalled maternal care (PBI –Care) and recalled maternal protection (PBI – Overprotection), and adolescent attachment insecurity (CAI). CAI was coded such that secure = 1 and insecure = 2

To address Aim 2, mediation analyses were conducted in MPlus using the sub-sample of participants (*n* = 210) who participated in the study while the PBI was part of the battery. The mother’s IIP score was entered as the independent variable and, depending on bivariate findings, the mother’s score on PBI recalled maternal care or overprotection was entered as the mediator, and a dichotomous CAI classification (secure vs. insecure or not disorganized vs. disorganized) was entered as the dependent variable. Age and gender were examined as possible covariates of attachment variables and were included as control variables if relevant. Path analysis (a form of structural equation modeling that uses observed variables) with exogenous variables was used to test mediation. Due to bivariate results, we tested the hypothesized mediation model shown in Fig. 2, with CAI insecure/secure attachment as the dependent variable (*y*), mother’s IIP total scores as the independent variable (*x*), and mother’s PBI recalled maternal care as the mediator variable (*m*). To evaluate mediation in this model, MPlus estimated bootstrapped confidence intervals (*n* = 5000 bootstrapped samples) using 95% bias-corrected confidence intervals. Of the 210 participants, 17 were missing data, which was accounted for using full information maximum likelihood. Due to missing data from three participants on the mediator variable (PBI), the Monte Carlo integration was employed. Deidentified data and MPlus syntax are available: https://osf.io/6zum4/?view_only=623a89db21914369b36fb95508d2e276.

## Results

### Preliminary analyses and aim 1 results: relations between maternal interpersonal problems and adolescent attachment

Skewness and kurtosis statistics (within range of − 1 to 1) revealed that continuous variables (IIP-SC and PBI scales) were normally distributed and could be evaluated using parametric tests. Regarding attachment classifications, 31.7% (*n* = 111) of adolescents were rated on the CAI as having secure attachment to their mothers, and 68.3% (*n* = 240) were rated as insecure using the two-way secure vs. insecure classification. Of the 240 insecurely attached adolescents, 46% (*n* = 161) were rated as dismissing, and 22.3% (*n* = 79) were rated as preoccupied using the three-way classification. On the disorganization scale, 86.6% (*n* = 303) were classified as not disorganized and 13.4% (*n* = 47) were classified as disorganized. All adolescents classified as disorganized were also classified as insecure.

**Fig. 3:**
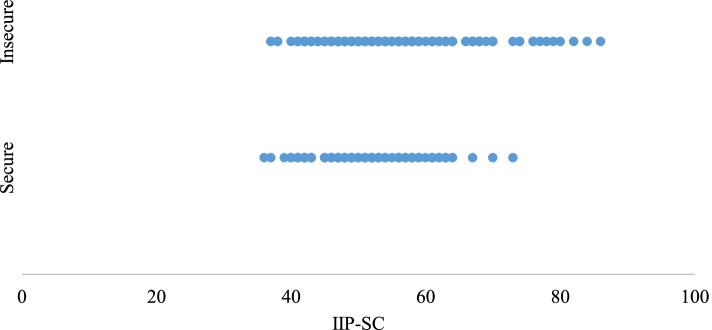
Categorical scatter plot of the relationship between adolescent attachment security (CAI) and maternal interpersonal problems (IIP-SC)

Binary logistic regressions were used to evaluate Aim 1. Higher maternal IIP scores were significantly associated with adolescent insecure attachment using the two-way classification (*b* = .04, *SE* = .01, *p* < .01, *OR* = 1.04). The relationship between maternal IIP and child attachment is displayed in Fig. 3. There was no significant association between maternal IIP and the not disorganized vs. disorganized classification. Results regarding the three-way secure vs. dismissing vs. preoccupied classification are in the supplementary materials. Based on these findings, we chose to further examine the relationship between maternal IIP and adolescent attachment using the two-way (secure vs. insecure) classification system in Aim 2. To inform subsequent analyses, child age and gender were tested as possible covariates with secure vs. insecure attachment. A chi-square test revealed no significant differences in gender and a *t*-test revealed no significant difference in age. Therefore, age and gender were not included in the subsequent analyses.

Regarding bivariate relationships between other main study variables, Pearson’s correlations revealed that interpersonal problems in mothers were associated with significantly lower levels of recalled care by their own mothers (*r* = −.26, *p* < .001) but not recalled overprotection. T-tests comparing the secure vs. insecure two-way classification groups revealed that mothers of adolescents with insecure attachment also had significantly lower levels of recalled maternal care than mothers of adolescents with secure classifications (*M =* 21.55 vs. 26.08*, t*(112.38) = 2.79, *p* < .01) but did not differ in recalled maternal overprotection. There were no significant differences between mothers of disorganized and not disorganized adolescents on recalled maternal care or recalled maternal overprotection.

### Aim 2 results: does mothers’ recalled maternal care mediate the relationship between maternal interpersonal problems and child attachment?

**Fig. 4:**
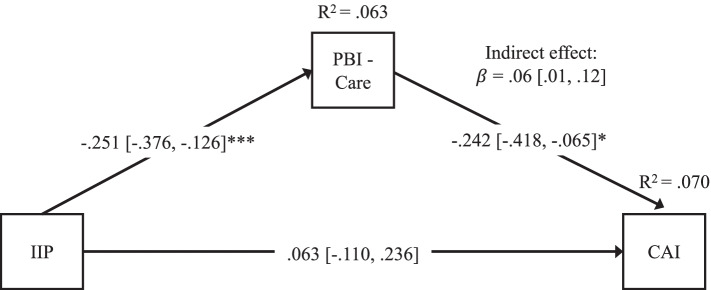
Path analysis mediation model results testing mothers’ recalled maternal care (PBI – care) as a mediator of the association between maternal interpersonal problems (IIP) and adolescent attachment insecurity (CAI). Values are standardized path coefficients with 95% confidence intervals

Based on bivariate findings, we tested one mediation model, with the mother’s recalled maternal care as mediator and adolescent secure vs. insecure attachment as the dependent variable. Standardized estimates are reported in Fig. 4. Mediation analyses revealed significant direct effects of maternal interpersonal problems on mothers’ recalled maternal care (*b* = −.30, *p* < .001, 95% CI [−.44, −.15]) and mothers’ recalled maternal care on offspring attachment security (*b* = −.05, *p* = .01, 95% CI [−.08, −.01]). The direct effect of maternal interpersonal problems on offspring attachment was nonsignificant (*b* = .01, *p* = .49, 95% CI [−.02, 06]). However, there was a significant indirect effect of maternal interpersonal problems on offspring attachment via the mother’s recalled maternal care (*b* = .01, *p* = .04, 95% CI [.003, 03]), such that greater levels of maternal interpersonal problems were associated with insecure adolescent attachment via lower levels of the mothers’ recalled maternal care. Together, maternal interpersonal problems and the mother’s recalled maternal care accounted for 7.0% of the variance in offspring attachment (*R*^*2*^ = .070).

## Discussion

Understanding whether maternal interpersonal problems, a key characteristic of personality pathology, are related to offspring attachment and mechanisms within this relationship would provide important opportunities for prevention and intervention considering the host of maladaptive outcomes associated with attachment insecurity, including offspring personality pathology [50, 56]. We hypothesized that 1) maternal interpersonal problems would be associated with insecure attachment in adolescent offspring and 2) the relationship between maternal interpersonal problems and adolescent attachment would be mediated by mothers’ own recalled maternal bonding.

In support of our first hypothesis, we found that greater interpersonal problems in mothers were associated with insecure attachment styles using the CAI two-way (secure vs. insecure) and three-way (secure vs. dismissing vs. preoccupied) attachment classifications. There was no association between maternal interpersonal problems and adolescent disorganized (vs. not disorganized) attachment classification. Given that all adolescents with a disorganized classification were also rated as insecure, differences in mothers of disorganized adolescents and other insecure adolescents may be more subtle, leading to non-significant findings. Additionally, unlike the secure vs. insecure classification, previous research has failed to find associations between the CAI disorganized attachment classification and self-report measures of attachment in adolescents [66], raising questions about this dichotomous rating scale during adolescence. These findings align with previous IPC research showing associations between interpersonal problems and maladaptive attachment in adult close relationships [22] and add to only one existing study on the intergenerational risk associated with parental interpersonal problems [28], which found that parental interpersonal problems were associated with adolescent BPD features. This is the first study to suggest a relationship between maternal interpersonal problems and offspring attachment.

Other notable bivariate findings include an association between the mothers’ interpersonal problems and lower recalled maternal care. Additionally, mothers of insecurely attached adolescents recalled lower levels of care by their own mothers. This aligns with other studies using the PBI, which have found that parent’s recalled parental bonding is associated with self-reported attachment-related feelings with offspring during pregnancy [21, 62] and at one and six months postpartum [20], as well as observed attachment behaviors 2 days following birth [62]. However, this is the first study to show associations between mothers’ recalled maternal bonding and attachment measured during adolescence. Unlike recalled maternal care, recalled maternal overprotection was not significantly associated with maternal interpersonal problems or offspring attachment.

Regarding our second hypothesis, mediation analyses revealed that maternal interpersonal problems were indirectly related to adolescent offspring attachment security via the mother’s recalled maternal care. This suggests that while maternal interpersonal problems and adolescent attachment security are related, the mother’s recalled experiences with their own caregivers may be an important driver within this relationship. It is important to note that the effect sizes were small, with maternal interpersonal problems and mother’s recalled maternal care together accounting for only 7.0% of the variance in offspring attachment. However, it is typical to expect a small association in cross-generational attachment research [67, 76], given the complex, multifactorial, and transactional nature of developmental processes, and this does not imply that the findings are not clinically meaningful. Future studies should examine other relevant variables such as maternal psychopathology or trauma history which may contribute significantly to the amount of variance in child attachment explained by regression models.

Regarding clinical applications of these findings, we focus on a population for which interpersonal problems are highly relevant: parents with personality pathology. While we did not directly measure parental personality pathology, there is theoretical and empirical support suggesting that interpersonal problems on the IPC aligns with severity of personality dysfunction as defined by the alternative model of personality disorders (AMPD; [28, 75]). Our results provide important new contributions to the literature on parents with personality disorders, which has focused primarily on borderline personality disorder (BPD) and has found that maternal BPD is associated with disrupted attachment in offspring [23, 24, 35, 36]. However, these studies relied on categorical DSM-5 Section II approaches to measuring personality pathology. We believe that measuring interpersonal problems provides a finer grained characterization of the processes that should be targeted in work with mothers to interrupt the intergenerational transmission of psychopathology. Additionally, existing research on maternal BPD and offspring attachment has been largely limited to dyads with infants [24, 35] or children [36]. The only study on maternal BPD and adolescent offspring attachment [23] used a self-report measure of offspring attachment, and no studies have used the CAI.

There have been widespread calls to understand mechanisms of intergenerational transmission and tailor intervention and prevention with dyads with parents with high levels of BPD [13] and IPC research methods and measures present exciting opportunities for future studies in this area. For example, researchers could use Event-Contingent Recording (ECR) of interpersonal behavior over periods of days or weeks or Continuous Assessment of Interpersonal Dynamics (CAID; [74]) to understand moment-to-moment parenting behaviors during in-vivo interactions. Examining parent-child interactions in dyads with high levels of parental interpersonal problems or personality pathology using such methods could further identify processes that lead to intergenerational transmission of risk and inform treatment. Moreover, researchers could also consider applying interpersonal and IPC-informed psychotherapy approaches [9, 74] to interventions for mothers with personality pathology or mothers of adolescents with attachment problems.

Our findings also suggest that attachment-based interventions (e.g., [60]) that consider the mother’s recollection of their attachment figures may be particularly helpful for mothers with interpersonal problems or personality pathology. This aligns with previous research showing that adult attachment style is highly related to interpersonal problems [22] and personality pathology [34]. Attachment-based interventions have shown promise for improving attachment between mothers with other psychopathology, such as depression (e.g., [65]) and substance abuse (e.g. [61]), and their offspring. While most of these interventions focus on dyads with infants and preschoolers, our findings add empirical justification for more deliberate treatment development of attachment-based interventions for adolescents and their caregivers. Most interventions that have integrated parenting into treatment for adults with personality pathology are largely skills-based and psychoeducational and do not directly address the attachment relationship [38, 52, 72]. Mentalization-based treatment (MBT; [15]), an evidence-based treatment that is grounded in attachment theory and considers the parent’s caregiving experiences, has been adapted for parents with BPD (MBT-P; [42]) and for families at risk of child maltreatment [8]. While there is an emerging evidence base for these interventions in dyads with young children, there is a need for further evaluation of their efficacy for dyads with adolescents. Moving forward, researchers should develop, adapt, and evaluate treatments that simultaneously address the attachment relationship and modify behaviors in parents with interpersonal problems or personality pathology.

The current study contains several limitations. Using multi-generational report (i.e., mother self-report of interpersonal problems and recalled caregiving experiences, and interview-based attachment assessment in adolescents) allowed us to evaluate mechanistic hypotheses; however, the cross-sectional design ultimately prohibits causal interpretation which can be fully obtained only through prospective follow-up studies. We also acknowledge that we could have tested an alternative perspective that recalled maternal bonding affects the strength and nature of the relationship between maternal interpersonal problems and child attachment, suggesting moderation analysis. While our mediation model reflects our study rationale that recalled maternal bonding is a proximal process that partially explains the intergenerational relationship between maternal interpersonal problems and adolescent attachment security, future research might consider testing this alternative model. It is also important to note that the PBI is a measure of recalled parental bonding and should not be viewed as representing the adults’ actual caregiving experiences. Retrospective reports of childhood memories are vulnerable to recall biases [4, 53] and may be influenced by other factors such as mood state, personality traits, and stressful life events [16, 54]. Therefore, the current study cannot draw conclusions regarding the links between actual caregiving experiences and adolescent offspring attachment. However, recalled childhood experiences can still impact how a parent interacts with their child when the parent’s *perception* of their experiences influences their parenting behaviors. Additionally, the perception or representation of attachment figures and how this perception impacts parenting, regardless of the individuals’ actual childhood experiences, can be considered and addressed in psychotherapy. Given recalled childhood experiences are more closely related to current psychopathology than prospectively measured childhood experiences [4, 11], it can even be argued that recalled bonding is a more relevant variable for the current study.

The composition of our sample presents further limitations. Participants consisted of predominantly white, non-Hispanic families seeking treatment at a private psychiatric hospital, and therefore findings may not generalize to families with different demographic characteristics. The homogenous nature of our sample also prohibited us from examining the role of demographic and socio-economic variables. Further, as an inpatient sample, adolescents displayed disproportionately high rates (63%) of insecure attachment, which raises the question of whether results would extend to non-psychiatric or community samples. Given that different measures of attachment must be used for younger children and based on the idea in attachment theory that relationship quality is gradually formed through dyadic interactions over time, the current findings may also not extend to dyads with younger offspring. Given the adolescent treatment setting, we did not collect full diagnostic information on the parents and were unable to examine personality disorder diagnoses or control for other parental psychopathology such as depression, which is also known to increase risk of insecure attachment in offspring [68]. Future studies should examine parental psychopathology as a potentially moderating or confounding variable in this model. Our conclusions are also limited to mothers and recalled maternal bonding. We excluded fathers because our hypotheses were based on previous research on attachment and parental bonding, which has focused on mothers, and because of the small sample size of father-report data using these variables that is available in our study. However, paternal interpersonal problems, recalled paternal bonding, and attachment to fathers are important areas for future research worthy of a full, separate evaluation.

Additionally, the CAI exhibited only moderate interrater reliability. This is in contrast to previous psychometric studies of the CAI [58, 66] and the related Adult Attachment Interview [3, 51], which report higher levels of interrater reliability. The attachment measures used in existing studies showing a relationship between maternal PBI and offspring attachment also demonstrated greater reliability; however, these measures were limited to mother report of infant attachment [21] or a non-established observational measure [62]. Therefore, while the use of an interview measure of child/adolescent attachment is a strength, further work should be done to replicate these findings, including studies with different measures of attachment. Moreover, only a subset of mothers from our larger sample completed the PBI and were therefore included in our mediation analysis. This subsample exhibited lower rates of depressive and anxiety disorders. However, there is no evidence to suggest that this difference between samples impacted the results.

## Conclusions

Withstanding limitations, our study has notable strengths. This is the first study to demonstrate links between maternal interpersonal problems on the IPC and attachment reported by adolescent offspring and between mothers’ recalled maternal bonding and adolescent attachment. Notably, we found these relationships using a large, clinical sample of adolescents and an interview measure of attachment. Findings suggest that while maternal interpersonal problems and adolescent attachment security are related, the mother’s own experiences with caregivers may be an important driver within this relationship. Therefore, attachment- or mentalization-based interventions that include consideration of how mothers recall their experiences with their own caregivers may be particularly relevant for interrupting the intergenerational transmission of risk. Additionally, researchers could consider drawing upon IPC measures and IPC-informed psychotherapy approaches to further examine mechanisms of intergenerational risk and to tailor interventions aimed to improve parent-child relations and attachment.

## Supplementary Information

**Additional file 1.**

## Data Availability

Data is not available in a public repository. Please contact the authors for further information on data and materials.
