# Supplementary material for: Maternal interpersonal problems and attachment security in adolescent offspring
Source: Borderline Personal Disord Emot Dysregul. 2022 Jul 1;9:18. doi: 10.1186/s40479-022-00188-8 (PMC9248194; doi:10.1186/s40479-022-00188-8)
Supplement: Supplementary file 1 — Additional file 1. [file 40479_2022_188_MOESM1_ESM.docx]

Supplementary material

**Results – three-way secure vs. dismissing vs. preoccupied classification**

A multinomial logistic regression revealed that maternal IIP scores were also significantly associated with the adolescent three-way attachment classification (*x^2^* = 8.90, *p* < .05). Using secure attachment as the reference category, higher maternal IIP scores were significantly associated with greater likelihood of dismissing (*b* = .03, *SE* = .02, *OR* = 1.03, *p* < .05) and preoccupied classifications (*b* = .05, *SE* = .02, *OR* = 1.05, *p* < .01).

The three-way attachment classification groups also differed on recalled maternal care (*F*(2, 186) = 3.84, *p* < .05). Tukey’s HSD tests revealed that mothers of secure adolescents reported significantly higher recalled maternal care than dismissing adolescents (p < .05), but no differences between mothers of secure vs. preoccupied or dismissing vs. preoccupied adolescents. The three groups did not differ on recalled maternal overprotection.
